# Phytochemical Analysis and Biological Activities of the Ethanolic Extract of *Daphne sericea* Vahl Flowering Aerial Parts Collected in Central Italy

**DOI:** 10.3390/biom11030379

**Published:** 2021-03-03

**Authors:** Claudio Frezza, Alessandro Venditti, Daniela De Vita, Fabio Sciubba, Pierpaolo Tomai, Marco Franceschin, Mirella Di Cecco, Giampiero Ciaschetti, Antonella Di Sotto, Annarita Stringaro, Marisa Colone, Alessandra Gentili, Mauro Serafini, Armandodoriano Bianco

**Affiliations:** 1 Dipartimento di Biologia Ambientale, Università di Roma “La Sapienza”, Piazzale Aldo Moro 5, 00185 Rome, Italy; daniela.devita@uniroma1.it; 2 Dipartimento di Chimica, Università di Roma “La Sapienza”, Piazzale Aldo Moro 5, 00185 Rome, Italy; alessandro.venditti@gmail.com (A.V.); fabio.sciubba@uniroma1.it (F.S.); pierpaolo.tomai@uniroma1.it (P.T.); marco.franceschin@uniroma1.it (M.F.); alessandra.gentili@uniroma1.it (A.G.); 3 NMR-Based Metabolomics Laboratory, Università di Roma “La Sapienza”, Piazzale Aldo Moro 5, 00185 Rome, Italy; 4 Ufficio Monitoraggio e Conservazione della Biodiversità Vegetale, Parco Nazionale della Majella, Via Badia 28, 67039 Sulmona, Italy; mirella.dicecco@parcomajella.it (M.D.C.); giampiero.ciaschetti@parcomajella.it (G.C.); 5 Dipartimento di Fisiologia e Farmacologia “V. Erspamer”, Università di Roma “La Sapienza”, Piazzale Aldo Moro 5, 00185 Rome, Italy; antonella.disotto@uniroma1.it; 6 Centro Nazionale per la Ricerca e la Valutazione Preclinica e Clinica dei Farmaci, Istituto Superiore di Sanità, Viale Regina Elena 299, 00161 Rome, Italy; annarita.stringaro@iss.it (A.S.); marisa.colone@iss.it (M.C.)

**Keywords:** *Daphne sericea* Vahl, Thymelaeaceae, phytochemical analysis, chemotaxonomy, polyphenols, antioxidant activities, cancer cell cytotoxicity

## Abstract

In this paper, the first phytochemical analysis of the ethanolic extract of *Daphne sericea* Vahl flowering aerial parts collected in Italy and its biological activities were reported. Eleven compounds were identified i.e., α-linolenic acid (**1**), tri-linoleoyl-*sn*-glycerol (**2**), pheophorbide *a* ethyl ester (**3**), pilloin (**4**), sinensetin (**5**), yuanhuanin (**6**), rutamontine (**7**), syringin (**8**), *p*-coumaric acid (**9**), *p*-anisic acid (**10**) and caffeic acid (**11**). To the best of our knowledge, compounds (**1**-**4**, **7-8** and **10**) were isolated from *D. sericea* for the first time during this work, whereas sinensetin (**5**) represents a newly identified component of the entire Thymelaeaceae family. The extract was found to possess radical scavenging against both DPPH^•^ and 2,2′-azino-*bis*(3-thylbenzothiazoline-6-sulfonic acid (ABTS^•+^) radicals, with at least a 40-fold higher potency against the latter. Moreover, chelating abilities against both ferrous and ferric ions have been highlighted, thus suggesting a possible indirect antioxidant power of the extract. Although the precise bioactive compounds remain to be discovered, the polyphenolic constituents, including phenolic acids, tannins and flavonoids, seem to contribute to the antioxidant power of the phytocomplex. In addition, the extract produced cytotoxic effects in MDA-MB-231 and U87-MG cancer cell lines, especially at the concentration of 625 μg/mL and after 48–72 h. Further studies are required to clarify the contribution of the identified compounds in the bioactivities of the extract and to support possible future applications.

## 1. Introduction

*Daphne sericea* Vahl is an evergreen shrub belonging to the Thymelaeaceae family. Its name derives from the union of two terms: the Greek noun “Δάφνη” (Dáphne), a mythological nymph who turned into a laurel plant, therefore related to the similarity of its leaves to those of laurel, and the Latin adjective “sericeus, a, um” meaning silky because of those silky hairs that cover the buds and the corolla tube [1].

**Figure 1:**
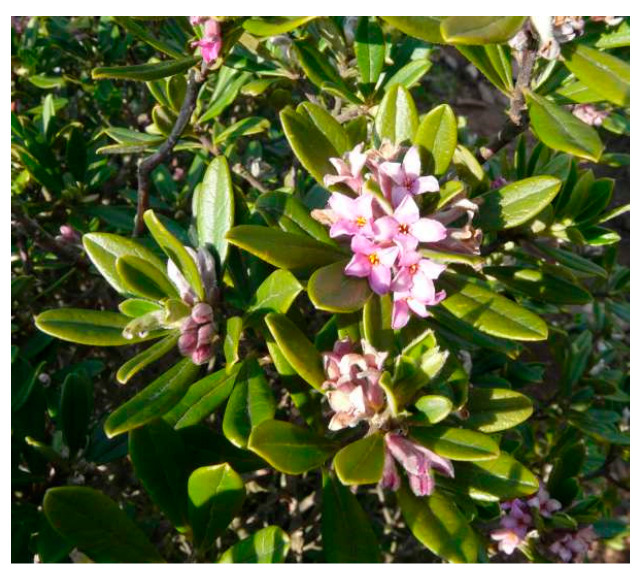
*Daphne sericea* Vahl.

From the morphological point of view, this species is characterized by erect or decumbent branches and coriaceous leaves, oblong–obovate, almost glabrous in the superior face and with appressed hairs beneath. The flowers are very fragrant, pink, mostly collected in terminal heads, blooming between February and May. The fruit is a reddish–brown drupe [2,3] (Figure 1).

This species has a Central–Eastern Mediterranean distribution, comprising Crete, Greece, Lebanon, Syria, Turkey and Italy [4] where it can be only found in Tuscany, Latium, Abruzzo, Molise, Campania, Apulia and Sicily [5]. Its typical habitat is represented by rocky slopes, open pinewoods and garigues up to 1000 m a.s.l. [3].

In Turkey, leaves and flowers of *D. sericea* have been used as traditional remedies for the treatment of hemorrhoids [6]; furthermore, branches with leaves are exploited as dye sources [7]. Similarly, other *Daphne* species are utilized in many areas of the world, especially in Asia, Africa and Europe, to treat several ailments, such as bruises, stomachache, infections, rheumatisms, diarrhea, inflammations, cancer, sore throat, indolent ulcers, snakebites, laryngitis, malaria, fever and apoplexy [8].

Only a few studies about the phytochemistry and pharmacological properties of *D. sericea* are available in the literature. Tongur et al. [9] studied the phytochemical composition of the acetone and methanolic extracts of *D. sericea* collected in Turkey and highlighted the presence of several flavonoids and organic acids (i.e., quercetin, luteolin, kaempferol, apigenin, isorhamnetin, rhamnetin, chlorogenic acid, caffeic acid, syringic acid, *p*-coumaric acid, ferulic acid, and rosmarinic acid). Moreover, these extracts were found to possess radical scavenging properties against DPPH^·^ and 2,2′-azino-*bis*(3-thylbenzothiazoline-6-sulfonic acid (ABTS^·+^) radicals, and against superoxide anion (O_2_^−^) [9]. Similarly, Ulubelen et al. [10] evaluated the phytochemical composition of a Soxhlet ethanolic extract from *D. sericea* collected in Turkey, reporting the presence of only flavonoids, including luteolin 7-methyl ether, isovitexin, apigenin and its 7-β-D-glucoside and two novel compounds, namely luteolin 7-methyl ether 5-β-D-glucoside and luteolin 7,3′-dimethyl ether 5-β-D glucoside. The aqueous layer remained after partitioning of the Soxhlet ethanolic extract with *n*-hexane, benzene and chloroform, mainly containing the coumarins daphnerotin and daphnin, sitosteryl 3-β-D-glucoside and several flavonoids (mainly luteolin 7-methyl ether, and lesser amounts of 5-glucoside of luteolin 7-methyl ether and 5-glucoside of luteolin 7,3′- dimethyl ether) exhibited promising antileukemic properties [10]. An antileukemic diterpenoid, namely mezerein, has been also identified in the hydroalcoholic extract of *Daphne mezereum* L. [11]. Lastly, 49 volatile compounds, mainly monoterpenes and fatty acids, representing 99.71 % of the total, were found in the essential oil of *D. sericea* flowers [12].

In the present study, a *D. sericea* specimen collected in Italy was studied for the first time for the phytochemical composition and the biological activities. Specifically, an ethanolic extract from the flowering aerial parts of *D. sericea*, has been prepared by maceration and characterized by an integrated phytochemical analysis. In addition, its antioxidant properties have been evaluated together with its cancer cell cytotoxicity for the first time.

## 2. Material and Methods

### 2.1. Plant Material

The plant material consisting of 350 g of flowering aerial parts was collected in the San Venanzio gorges, Raiano municipality, Abruzzo region, Italy (geographical coordinates 13°79′84″ E, 42°10′77″ N) at an altitude of 450 m a.s.l. in the month of April 2018. The botanical identification was performed by two of us (M.D.C and G.C.) through the most used analytical floras [2,3] and comparing the collected material with those conserved in the principal herbariums of Central Italy. A portion of this collection was stored in our laboratory for further references and registered under the voucher name DS15042018.

### 2.2. Chemicals

The following solvents and reagents were used during this study: ethanol absolute for the extraction procedure and for the dissolution of the extract for the antioxidant activity assays; methanol and chloroform in mixture at different concentrations as eluting systems for the separation procedure on silica gel (60 Å, 70–230 mesh) columns and as mobile phases for the TLCs; 2N H_2_SO_4_ for the development of the TLCs; CDCl_3_, CD_3_OD and D_2_O as solvents for the identification of the metabolites by NMR spectroscopy; HPLC grade methanol for the identification of the metabolites by mass spectrometry (MS); MTT (3-(4,5-dimethylthiazol-2-yl)-2,5-diphenyltetrazolium bromide) as an addition for the incubation of cells in the medium; DMSO for the dissolution of cells; Folin–Ciocalteu’s phenol reagent, tannic acid (Ph Eur purity), sodium carbonate (Na_2_CO_3_; 99.999% purity), aluminum chloride hexahydrate (AlCl_3_ × 6H_2_O; Ph Eur purity), 1,1-diphenyl-2-picryl-hydrazyl (DPPH^·^; 95% purity), 2,2′-azino-*bis*(3-thylbenzothiazoline-6-sulfonic acid) diammonium salt (ABTS^·+^; 98% purity), 2,2′-azo-*bis*(2-methylpropionamidine) dihydrochloride (AAPH; 97% purity), iron (II) sulfate heptahydrate (FeSO_4_ × 7H_2_O; 99% purity), iron (III) chloride (FeCl_3_ × 6H_2_O; 97% purity), ferrozine (97% purity), hydroxylamine hydrochloride (98% purity), trolox (97% purity) and quercetin (>95% purity) for the antioxidant activity assays.

All the solvents were RPE purity grade if not otherwise specified, and were purchased from Sigma Aldrich (San Louis, MI, USA) as well as all the deuterated solvents, 2N H_2_SO_4_, HPLC grade methanol and MTT, whereas silica gel, DMSO and all the reagents for the antioxidant activity assays were purchased from Merck (Darmstadt, Germany).

### 2.3. Extraction Procedure

The dried flowering aerial parts (350.2 g) were inserted into a flask and covered with a solution of 96% ethanol until complete immersion (about 300 mL). The plant material was macerated for at least 72 h so that metabolites could come into solution. This procedure was repeated three times in order to produce an exhaustive extraction. The ethanolic solutions, showing a greenish coloration, were gathered together and then filtered. All the ethanol was eliminated at reduced pressure at 50 °C until a water suspension was obtained. Throughout this part, pH was checked on litmus paper and this was about 8. This passage is necessary in order to verify that the pH is not too acid or basic (meaning in the range 5.5–8.5) because an extreme acidity or alkalinity might cause secondary reactions in the extract such as the hydrolysis of ester and glycosidic bonds [13]. The obtained suspension was then frozen and later lyophilized to preserve temperature-sensitive compounds eventually present. The obtained dried crude extract weighed 23.4 g and was a dark green color.

### 2.4. Isolation and Identification of the Metabolites

An aliquot of the dried crude extract (3.0 g) was subjected to a column chromatography procedure on silica gel as the stationary phase. The used amount of silica gel was 93.2 (ratio ~ 1:30 *w*/*w*) and the eluting system was a mixture of chloroform and methanol at different concentration ratios. The initial concentration ratio was 98:2 (*v/v*) (300 mL) but, during the chromatographic run, this was changed raising the polarity in order to allow the elution of the most polar compounds too. In particular, the concentration ratio passed to 95:5 (*v/v*) (300 mL), 9:1 (*v/v*) (400 mL), 8:2 (*v/v*) (500 mL), 7:3 (*v/v*) (300 mL) and then to 6:4 (*v/v*) (200 mL). From this chromatographic procedure, all the compounds were identified by means of exhaustive comparison of their spectroscopic data with those reported in the literature and with authentic standard compounds already available in our laboratory. In particular, the identified compounds were the following: tri-linoleoyl-*sn*-glycerol (**2**) and pheophorbide *a* ethyl ester (**3**) [14,15] in mixture in a ratio of 20:1 *(w/w)* from the assembly of fractions 6–11 for the total weight of 10.2 mg; α-linolenic acid (**1**) and pilloin (**4**) [14,16] in mixture in a ratio of 4:1 (*w/w*) from the assembly of fractions 21–30 for the total weight of 16.9 mg; pilloin (**4**) and sinensetin (**5**) [16,17] in mixture in a ratio of 5:1 (*w/w*) from the assembly of fractions 31–33 for the total weight of 5.4 mg; pilloin (**4**) and rutamontine (**7**) [16,18] in mixture in a ratio of 1.5:1 (*w/w*) from the assembly of fractions 34–35 for the total weight of 7.7 mg; *p*-coumaric acid (**9**) [19] in mixture with other unidentified compounds (ratio not calculable) from the assembly of fractions 115–127 for the total weight of 12.1 mg; syringin (**8**) [15] in mixture with ethoxy glucose and several saccharides (ratio not calculable) from the assembly of fractions 138–147 for the total weight of 6.8 mg; yuanhuanin (**6**) [20] and *p*-anisic acid (**10**) [21] in mixture in ratio 10:1 (*w/w*) from the assembly of fractions 161–179 for the total weight of 6.6 mg; caffeic acid (**11**) [22] in mixture with several saccharides (ratio not calculable) from the methanol column wash for the total weight of 33.8 mg. The identification of components in mixture was conducted by applying a well-established method developed by our research group [23,24,25,26,27,28].

### 2.5. NMR Analysis

NMR spectra were recorded on a Bruker Avance III instrument (Billerica, MA, USA). The potency of the instrument was 400 MHz. The chemical shifts were expressed in ppm from TMS (s, 0.00 ppm) as internal reference standard for the spectra recorded in CDCl_3_, whereas the internal solvent signal of CD_2_HOD (m5, δ_H_ 3.31 ppm; m7, δ_C_ 49.00 ppm) was the reference for the spectra recorded in CD_3_OD and the HDO signal (s, 4.79 ppm) was set as reference for the spectra recorded in D_2_O. All the experiments were conducted at 298 K.

The 2D NMR spectra were performed on the same Bruker Avance III 400 MHz instrument operating at 9.4 T and 298 K. The Heteronuclear Single Quantum Coherence (HSQC) experiments were acquired with a spectral width of 15 ppm for the proton nucleus and 250 ppm for the carbon nucleus. The average direct coupling (^1^*J*_C-H_) was set to 145 Hz. The recycle delay was 2.0 s and the data matrix set was 4 K × 256 points. The Heteronuclear Multiple Bond Correlation (HMBC) experiments were acquired with the same spectral width applied in the HSQC experiments and by using two different values of long-range coupling constants of 8 Hz with a recycle delay of 2.0 s and a data matrix of 4 K × 256 points. NMR spectra were analyzed by ACD NMR manager software ver. 12 (ACD/Labs, Toronto, ON, Canada).

### 2.6. MS Analysis

MS spectra were acquired with a triple quadrupole mass spectrometer PE-Sciex API-3000^®^ (Perkin Elmer Sciex, Toronto, ON, Canada), equipped with an ESI source operating in positive ionization mode. The capillary voltage was 4500 V. High purity nitrogen was used as the curtain gas, while air was employed as the nebulizer and drying gas. The temperature to heat the drying gas was set at 100 °C. The flow rate of sample infusion was 20 μL/min. MS spectra were acquired with 50 acquisitions per sample. The full width at half maximum (FWHM) was set at *m/z* 0.7 ± 0.1 in each mass-resolving quadrupole to operate with a unit resolution. The mass spectrometer operated in Full Scan mode in a mass spectral range of 100–1000 m/z. MS data were acquired and elaborated by Analyst^®^ 1.5 Software (AB Sciex, Framingham, MA, USA).

### 2.7. NMR and MS Data of the Isolated Compounds

α-linolenic acid (**1**): ^1^H NMR (400 MHz, CDCl_3_) δ: 5.42–5.32 (6H, m, overlapped olefinic protons), 2.82–2.75 (4H, m, H-11 and H-14), 2.36–2.30 (2H, m, H-2), 2.09–1.97 (4H, m, H-8 and H-17), 1.64–1.58 (2H, m, H-3), 1.25 (8H, br. s, H-4, H-5, H-6, H-7), 0.86 (3H, br. t, *J* = 7.2 Hz, 18-CH_3_).

ESI-MS: *m/z* 279.38 [M+H]^+^, *m/z* 301.41 [M+Na]^+^.

tri-linoleoyl-*sn*-glycerol (**2**): ^1^H NMR (400 MHz, CDCl_3_) δ: 5.42–5.36 (12H, m, overlapped olefinic protons), 5.34–5.31 (1H, partially overlapped, H-b), 4.17–4.09 (4H, m, H-a, H-c), 2.82–2.75 (6H, m, H-11, H-11′ and H-11”), 2.36–2.26 (6H, m, H-2, H-2′ and H-2”), 2.09–2.00 (12H, m, H-8, H-8′, H-8”, H-13, H-13′ and H-13”), 1.62–1.56 (6H, m, H-3, H-3′ and H-3”), 1.25 (52H, br. s, n-(CH_2_)), 0.89–0.85 (9H, m, 18-CH_3_, 18′-CH_3_, 18″-CH_3_).

ESI-MS: *m/z* 901.95 [M+Na]^+^.

pheophorbide *a* ethyl ester (**3**): ^1^H NMR (400 MHz, CDCl_3_) δ: 9.69 (1H, s, H-10), 9.54 (1H, s, H-5), 8.72 (1H, s, H-20), 8.01 (1H, dd, *J* =17.9, 11.5 Hz, H-3′), 6.22 (1H, dd, *J* = 11.5, 1.3 Hz, H-3”), 3.88 (3H, s, H-13^IV^), 3.72 (3H, s, H-12′), 3.43 (3H, s, H-2′), 3.27 (3H, s, H-7′), 1.87 (3H, s, H-18′), 1.12 (3H, t, *J* = 7.1 Hz, H-17^V^).

ESI-MS: *m/z* 643.68 [M+Na]^+^.

pilloin (**4**): ^1^H NMR (400 MHz, CDCl_3_) δ: 7.49 (1H, dd, *J* = 8.5, 2.0 Hz, H-6′), 7.33 (1H, d, *J* = 2.0 Hz, H-2′), 7.04 (1H, d, *J* = 8.5 Hz, H-5′), 6.58 (1H, s, H-3), 6.49 (1H, d, *J* = 2.2 Hz, H-8), 6.37 (1H, d, *J* = 2.2 Hz, H-6) 4.01 (3H, s, 4′-OMe), 3.89 (3H, s, 7-OMe).

ESI-MS: *m/z* 337.31 [M+Na]^+^, *m/z* 313.29 [M-H]^−^.

sinensetin (**5**): ^1^H NMR (400 MHz, CDCl_3_) δ: 7.57 (1H, dd, *J* = 8.4, 2.0 Hz, H-6′), 7.37 (1H, d, *J* = 2.0 Hz, H-2′), 7.02 (1H, d, *J* = 8.4 Hz, H-5′), 6.72 (1H, s, H-3), 6.62 (1H, s, H-8), 3.99 (3H, s, 4′-OMe), 3.98 (3H, s, 3′-OMe), 3.97 (3H, s, 6-OMe), 3.95 (3H, s, 7-OMe).

ESI-MS: *m/z* 395.38 [M+Na]^+^, *m/z* 371.40 [M-H]^−^.

yuanhuanin (**6**): ^1^H NMR (400 MHz, CD_3_OD) δ: 7.34 (1H, overlapped, H-6′), 7.33 (1H, overlapped, H-2′), 6.88 (1H, d, *J* = 9.0 Hz, H-5′), 6.86 (1H, d, *J* = 2.4 Hz, H-6), 6.82 (1H, d, *J* = 2.4 Hz, H-8), 4.86 (1H, d, *J* = 8.0 Hz, H-1″), 3.93 (1H, d, *J* = 12.0 Hz, H_a_-6″), 3.91 (3H, s, 7-CH_3_O), 3.84 (1H, d, *J* = 12.0 Hz, H_b_-6″), 3.70 (1H, m, H-3″*), 3.64 (1H, m, H-2″), 3.54 (1H, m, H-5″*), 3.44 (1H, m, H-4″).

^13^C-NMR (100 MHz, CD_3_OD) δ: 180.3 (C-4), 166.0 (C-7), 164.6 (C-2), 160.5 (C-9), 159.7 (C-5), 150.8 (C-4′), 147.0 (C-3′), 123.3 (C-1′), 120.2 (C-6′), 116.8 (C-5′), 114.1 (C-2′), 110.3 (C-10), 106.7 (C-3), 104.9 (C-1″), 104.1 (C-6), 97.4 (C-8), 77.5 (C-5″*), 76.7 (C-3″*), 71.7 (C-2″), 69.3 (C-4″), 62.6 (C-6″), 56.7 (O-Me).

* signals may be reversed.

ESI-MS: *m/z* 485.43 [M+Na]^+^, *m/z* 461.46 [M-H]^−^.

rutamontine (**7**): ^1^H NMR (400 MHz, CDCl_3_) δ: 7.68 (1H, d, *J* = 9.5 Hz, H-4′), 7.43 (1H, s, H-4), 7.41 (1H, d, *J* = 8.3 Hz, H-5′), 6.97(1H, br. d, *J* = 8.3 Hz, H-6′), 6.90 (1H, br. s, H-8′), 6.84 (1H, s, H-5), 6.35 (1H, d, *J* = 9.5 Hz, H-3′), 3.90 (3H, s, OMe).

ESI-MS: *m/z* 375.31 [M+Na]^+^, *m/z* 351.27 [M-H]^−^.

syringin (**8**): ^1^H NMR (400 MHz, CD_3_OD) δ: 6.73 (2H, s, H-2′, H-6′), 6.52 (1H, d, *J* = 15.9 Hz, H-3), 6.31 (1H, dt, *J* = 15.9, 5.6 Hz, H-2), 4.88 (1H, overlapped signal, H-1”), 4.22 (2H, d, *J* = 5.2 Hz, H-1), 3.84 (6H, s, 2xOMe), 3.75 (1H, dd, *J* = 12.1, 2.2 Hz, Ha-6”), 3.64 (1H, dd, *J* = 12.1, 5.1 Hz, Hb-6”), 3.57-3.40 (3H, m, H-3”, H-4”, H-5”), 3.30-3.26 (1H, m, H-2”).

ESI-MS: *m/z* 395.36 [M+Na]^+^, *m/z* 411.42 [M+K]^+^, *m/z* 767.58 [2M+Na]^+^.

*p*-coumaric acid (**9**): ^1^H NMR (400 MHz, CD_3_OD) δ: 7.59 (1H, d, *J* = 16.0 Hz, H-β), 7.44 (2H, d, *J* = 8.5 Hz, H-2 and H-6), 6.80 (2H, d, *J* = 8.5 Hz, H-3 and H-5), 6.28 (1H, d, *J* = 16.0 Hz, H-α).

ESI-MS: *m/z* 187.15 [M+Na]^+^, *m/z* 163.08 [M-H]^−^.

*p*-anisic acid (**10**): ^1^H NMR (400 MHz, CD_3_OD) δ: 7.86 (2H, d, *J* = 8.8 Hz, H-3, H-5), 6.93 (2H, d, *J* = 8.8 Hz, H-2, H-6), 3.88 (3H, s, 4-OCH_3_).

ESI-MS: *m/z* 175.27 [M+Na]^+^.

caffeic acid (**11**): ^1^H NMR (400 MHz, D_2_O) δ: 7.56 (1H, d, *J* = 15.9 Hz, H-β), 7.05 (1H, br. s, H-2), 6.92 (1H, br. d, *J* = 8.3 Hz, H-6), 6.71 (1H, d, *J* = 8.3 Hz, H-5), 6.29 (1H, d, *J* = 15.9 Hz, H-α).

ESI-MS: *m/z* 203.09 [M+Na]^+^, *m/z* 179.10 [M-H]^−^.

### 2.8. Antioxidant Activity

Different antioxidant activities, including radical scavenging, chelating and reducing ones, were assessed according to previous standardized spectrophotometric methods, using a microplate reader (Epoch Microplate Spectrophotometer, BioTeK^®^ Instruments Inc., Winooski, VT, USA) [29]. Particularly, the radical scavenger activity towards the synthetic DPPH^·^ (1,1-diphenyl-2-picryl-hydrazyl) and ABTS^·+^ (2,2′-azino-*bis*(3-thylbenzothiazoline-6-sulfonic acid) diammonium salt) radicals, and the ability of the extract to indirectly interfere with the reactive oxygen species (ROS)-generation through blocking the Fenton reaction by iron (both ferrous and ferric ions) chelating and reducing effects, were evaluated. The antioxidant properties were correlated with the polyphenol content of the extract. To this end, the total amounts of phenolics, tannins and flavonoids were measured and expressed by the microplate Folin–Ciocalteu and aluminum trichloride methods, described by Di Sotto et al. [30], and expressed as tannic acid (TAE) and quercetin equivalents (QE) per mg of extract, respectively.

To perform the antioxidant activity assays, *D. sericea* extract was dissolved in 100% EtOH (*v/v*) and assayed in the concentration range of 1–2000 µg/mL, using suitable dilution factors in order to achieve a concentration–response curve. Data obtained from at least two experiments and six technical replicates for each concentration were pooled for the statistical analysis. In each experiment, negative (vehicle) and positive controls (standard antioxidant agents) were included. Particularly, trolox (concentration range 0.1–100 µg/mL) and quercetin (concentration range 1–2000 µg/mL) were used as positive controls for the radical scavenger and reducing activity, and for the chelating activity, respectively. Moreover, suitable controls of the possible interference absorbance of the extract were included.

### 2.9. Cytotoxic Activity

HDF (human dermal fibroblast) cells, MDA-MB-231 (human breast cancer cells) and U-87 MG (human brain glioblastoma-astrocytoma cell lines) were obtained from ATCC, Manassas, VA, USA. HDF and MDA-MB-231 cells were grown in Dulbecco’s Modified Eagle’s Medium (DMEM) medium whereas U-87 MG was grown in DMEM/F12 medium with 10% fetal bovine serum (HyClone™ Fetal Bovine Serum (U.S.), Characterized) and 100 units/mL of penicillin/streptomycin at 37 °C in a humidified atmosphere in 5% CO_2_ incubator.

The HDF, MDA-MB-231 and U-87 MG cell lines were seeded into 96-well microtiter plates (Corning^®^, Saint Louis, MO, USA) at a density of 1.2*10^4^, 1.0*10^4^ and 1.5*10^4^ cells/wells, respectively. After overnight incubation, cells were exposed to increased concentrations of the ethanolic extract of *D. sericea* Vahl (125, 625 and 1.25 mg/mL) in cell culture medium. After incubation times of 24, 48 and 72 h, medium was replaced by a new fresh one containing 0.5mg/mL of MTT. After 2 h, at 37°C in 5% CO_2_ cells were dissolved in DMSO. Absorbance was read at 570 nm by a spectrophotometer plate reader. The data were expressed as absorbance relative to untreated cells in the same experiment and standardized to 100%. All data points were performed in triplicate and in, at least, three independent experiments.

### 2.10. Statistical Analysis

For the antioxidant activity studies, data are reported as the average and standard error of at least six replicates from two experiments. Statistical analysis was performed with GraphPadPrism™ software (GraphPad Software Inc., San Diego, CA, USA).

Differences among treatments were evaluated by one-way analysis of variance (one-way ANOVA), followed by Dunnett’s multiple comparison posttest. A *p* < 0.05 was considered as significant. The “Hill equation”: E = E_max_/ 1 + (10 ^LogEC50^/A)^HillSlope^, where E is the effect at a given concentration, E_max_ is the maximum activity, IC_50_ is the concentration producing a 50% inhibitory response, A is the agonist concentration, and Hill Slope is the slope of the agonist, was applied to obtain a concentration–response curve. The Pearson correlation coefficient was used to calculate the correlation between two variables, while statistical significance was determined by the two-tailed *t*-test.

For the cytotoxic activity studies, the normally distributed data were reported as the mean ± standard deviation and compared using analysis of variance (ANOVA). All experiments were quadruplicate; *p* < 0.05 was considered statistically significant.

## 3. Results and Discussion

### 3.1. Phytochemistry

**Figure 2:**
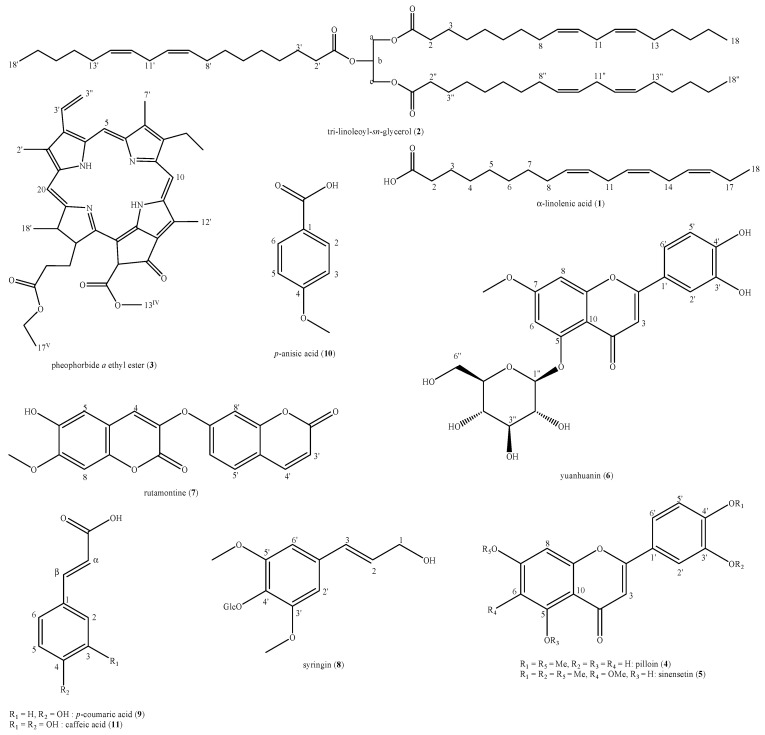
Structures of the identified compounds in *Daphne sericea* Vahl flowering aerial parts.

The phytochemical analysis of the *D. sericea* flowering aerial parts collected in central Italy led to the identification of eleven compounds: α-linolenic acid (**1**), tri-linoleoyl-*sn*-glycerol (**2**), pheophorbide *a* ethyl ester (**3**), pilloin (**4**), sinensetin (**5**), yuanhuanin (**6**), rutamontine (**7**), syringin (**8**), *p*-coumaric acid (**9**), *p*-anisic acid (**10**) and caffeic acid (**11**) (Figure 2).

These compounds belong to six different major classes of natural compounds i.e., fatty acids and derived triglycerides (**1,2**), tetrapyrrole derivative of chlorin family, also considered as degradation product of chlorophylls (**3**), flavonoids (**4–6**), coumarins (**7**), lignols (**8**) and natural organic acids (**9–11**).

### 3.2. Chemotaxonomy

To the best of our knowledge, compounds **1**–**4**, **7–8** and **10** have been identified in *D. sericea* for the first time, during this work. In addition, sinensetin (**5**) represents a newly identified component of the entire Thymelaeaceae family. Indeed, this compound is more typical of another species i.e., *Orthosiphon aristatus* (Blume) Miq. (Lamiaecae family) [31,32,33,34]. This result is very important from the chemosystematic point of view since Lamiaceae and Thymelaceae are quite distant families. Therefore, it is possible that the biogenetic pathway leading to compound (**5**) evolved independently in Thymeleaceae and Lamiaceae. Obviously, further studies are necessary to confirm this hypothesis. On the contrary, *p*-coumaric acid (**9**) and caffeic acid (**11**) have been already isolated in this species [8]. Pheophorbide *a* ethyl ester (**3**), which may also be an artifact due to the extraction methodology adopted, has been already isolated from *D. oleoides* Schreb. [15]. As for pillion (**4**), it has been reported before only in *D. aurantiaca* Diels [35]. Yuanhuanin (**6**) was originally recognized in *D. sericea* [8] and then isolated also from *D. koreana* Nakai and *D. pedunculata* H.F.Zhou ex C.Y.Chang (syn. of *D. esquirolii* subsp. *pedunculata* (H.F.Zhou ex C.Yung Chang) Halda) [36]. Moreover, rutamontine (**7**) is quite a rare compound of *Daphne* species. In fact, its occurrence has been reported only in *D. acutiloba* Rehder [37] and *D. oleoides* Schreb. [38]. Syringin (**8**) has been identified in *D. oleoides* Schreb. [15,38] but also in many other *Daphne* species like *D. arisanensis* Hayata [39], *D. kiusiana* var. *atrocaulis* (Rehder) F.Maek. [40], *D. tangutica* Maxim. [41], *D. mucronata* subsp. *linearifolia* (Hart) Halda. [42] and *D. feddei* H.Lév. [43], thus resulting a highly represented metabolite within the genus. Lastly, *p*-anisic acid (**10**) has been previously isolated from *Daphne* species only from *D. holosericea* (Diels) Hamaya [44].

### 3.3. Biological Activities

#### 3.3.1. Antioxidant Activity

**Figure 3:**
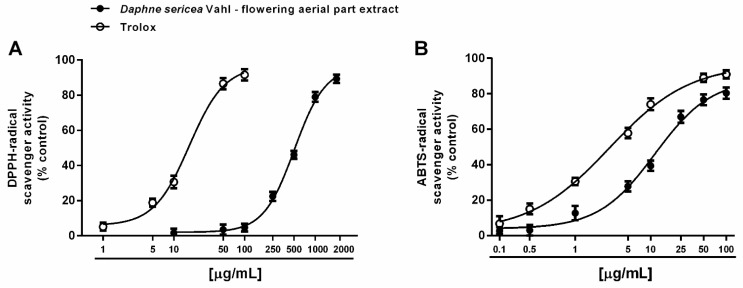
Scavenger activity of the ethanolic extract obtained from the flowering aerial parts of *Daphne sericea* Vahl and the positive control Trolox against DPPH^·^ (**A**) and ABTS^·+^ (**B**) radicals. Each data point represents the average and standard error of at least 6 replicates.

Both direct and indirect antioxidant properties of the ethanolic extract from the flowering aerial parts of *D. sericea* were evaluated. A direct antioxidant power has been assessed by measuring the ability of the extract to neutralize the synthetic DPPH^·^ and ABTS^·+^ radicals. Under our experimental conditions, *D. sericea* flowering aerial parts extract exhibited scavenging properties towards both radicals, although with a different potency. Indeed, it inhibited DPPH^·^, starting from 250 μg/mL achieving a maximum effect at the concentration of 2000 μg/mL (Figure 3A), while the inhibition of ABTS^·+^ radical already appeared at 5 μg/mL and became complete at 100 μg/mL (Figure 3B).

This different behavior was also confirmed by the IC_50_ values, which highlighted at least a 40-fold higher potency of the extract against ABTS^·+^ with respect to DPPH^·^ (Table 1). The ABTS^·+^ radical scavenging activity was four-fold lower than that of the positive control Trolox, with a marked 30-fold lower potency against DPPH^·^ (Table 1). The Pearson correlation analysis highlighted that these activities were not significantly correlated (Table 2).

Electron- or hydrogen-transfer mechanisms are required for scavenging both DPPH^·^ and ABTS^·+^ radicals, although with a different specificity and kinetic profile [45]. Indeed, DPPH^·^ is usually neutralized by small molecules, being sterically limited the access to the radical site to bulky compounds [45]. For instance, carotenoids have been reported to be not effective against DPPH^·^ [46]. Conversely, ABTS^·+^ can react with both lipophilic and hydrophilic molecules, with poor selectivity for hydrogen-atom donors, thus being widely applied to characterize the radical scavenging power of different herbal extracts and vegetables. Considering the different behavior of DPPH^·^ and ABTS^·+^ radicals and the specific ability of *D. sericea* extract against ABTS^·+^, the contribution of a complex pool of phytochemicals, including both lipophilic and hydrophilic, and either small or bulky structures, can be assumed; however, to confirm this hypothesis, further studies are required.

**Figure 4:**
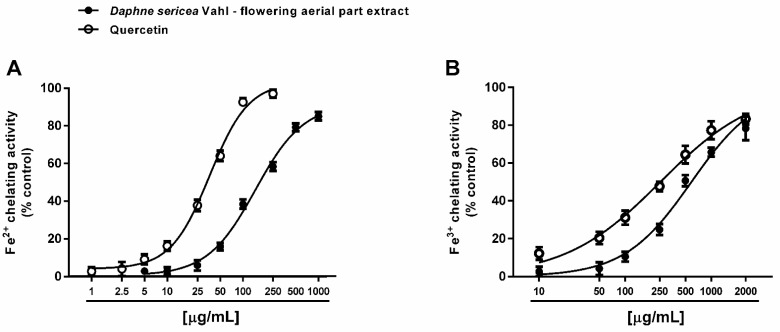
Chelating activity of the ethanolic extract obtained from the flowering aerial parts of *Daphne sericea* Vahl and the positive control quercetin against ferrous (**A**) and ferric (**B**) ions. Each data point represents the average and standard error of at least 6 replicates.

**Figure 5:**
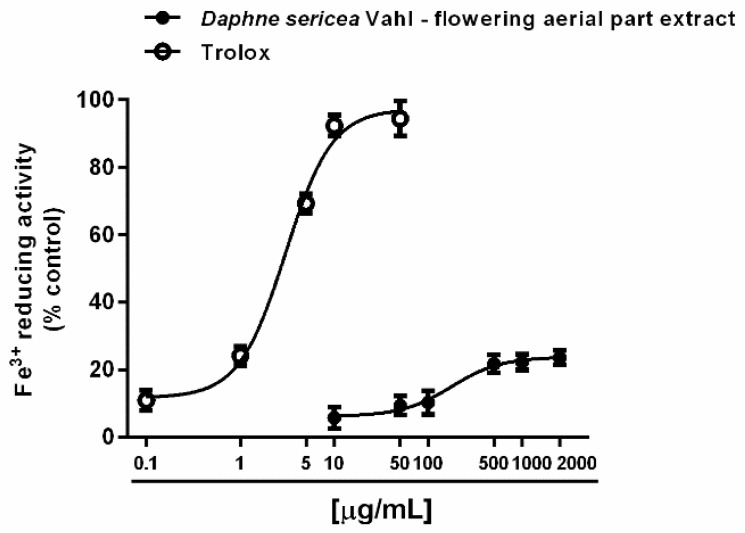
Ferric ion reducing activity of the ethanolic extract obtained from the flowering aerial parts of *Daphne sericea* Vahl and the positive control trolox. Each data point represents the average and standard error of at least 6 replicates.

Under our experimental conditions, *D. sericea* flowering aerial parts extract was also able to interact with both ferrous and ferric ions. Particularly, it exhibited significant chelating properties starting from the concentration of 50 μg/mL (Figure 4A,B), although with a low reducing efficacy (Figure 5).

Comparing the IC_50_ values, the ferrous ion chelating ability of the extract was almost four-fold more potent than that of ferric ions, but significantly lower than those of the positive control quercetin. As estimated by the Pearson analysis, the ferrous and ferric ion chelating activities resulted significantly correlated each other and with DPPH^·^ scavenging activity (at least *p* < 0.05) (Table 2).

Colorimetric determinations of polyphenols highlighted that the extract was characterized by a 3.4 polyphenols/tannins ratio, along with about 0.8% of total flavonoid compounds (Table 3).

The antioxidant properties of *D. sericea* Vahl extracts have been scantily investigated, despite a higher interest for different *Daphne* species [47,48,49,50]. Recently, Tongur et al. [9] evaluated the antioxidant properties of the methanolic and acetone leaf extracts of *D. sericea* (ESM and ESA, respectively) in relation to its polyphenolic constituents, highlighting that total flavonoids and polyphenols (121.3 ± 19.7 and 602.4 ± 23.6 μg per mg of extract, expressed as rutin and gallic acid equivalents, respectively) were mainly recovered by methanolic extraction. This composition was found to be associated to interesting scavenging properties of extracts against both DPPH^·^ and ABTS^·+^ radicals, with a higher potency of ESM. This evidence agrees with our findings, although our *D. sericea* ethanolic extract exhibited a more potent ABTS^·+^ scavenging activity (IC_50_ almost 14-fold lower than that of ESM), despite its eight-fold lower potency against DPPH^·^. Furthermore, Zengin et al. [51] characterized the antioxidant power of different extracts obtained from the leaves of the Turkish *D. serica* variety and highlighted that total flavonoids and polyphenols were mainly recovered by ethyl acetate and ethanol with respect to hexane and methanol; nevertheless, the methanolic extract exhibited higher potency as a DPPH^·^ radical scavenger. This evidence suggests that the extraction method is crucial to recover bioactive compounds and can be responsible for the differences in the extract potency. Indeed, it is known that several factors, including the origin of plant material, harvesting time, extraction conditions (e.g., solvent, temperature, pH, extraction time) and methodology (e.g., classical maceration, Soxhlet extraction), and solvent evaporation, can affect the phytochemical composition of an extract and its bioactivities [52]. Further studies could clarify if modifying extraction conditions is a suitable strategy for better recovering the bioactive constituents of *D. sericea* flowering aerial parts, thus enhancing its antioxidant power.

Under our experimental conditions, *D. sericea* extract also exhibited iron chelating activities despite a weak ferric reducing power. Despite our results, [51] found that all the extracts from *D. sericea*, especially the methanolic and ethanolic ones, were able to reduce both ferric ions, although with a lower potency with respect to the standard antioxidants BHA (butylated hydroxyanisole)and BHT (butylated hydroxytoluene). Conversely, to the best of our knowledge, the chelating activity of *D. sericea* has been not previously published.

Iron is involved in metal-catalyzed oxidation of biological substrates and in the generation of reactive oxygen species (ROS) species through the Fenton cascade. The species can oxidate cell macromolecules, such as DNA, RNA, phospholipid biomembrane and proteins, thus leading to different chronic diseases, such as neurodegeneration, diabetes, cardiovascular and metabolic ailments, and cancer [53,54,55]. Therefore, an increasing interest has been devoted to the identification of novel antioxidant agents to be exploited in the prevention and treatment of such diseases, although clinical evidence of benefits and bioavailability remain important issues to be considered [55]. Electron donating compounds and chelating agents can affect Fenton reaction and lower ROS generation, thus acting as indirect antioxidant agents [56].

Several polyphenols and flavonoids, especially ellagic acid, rutin and catechin have been reported to possess a marked chelating power, which seems to be responsible for their antioxidant and cytoprotective properties [57].

Among the identified compounds in *D. sericea* flowering aerial parts extract, pilloin (**4**) has been previously reported to possess antioxidant properties likely due to the presence of a catechol ring in the structure [58]. Similarly, antioxidant activities have been reported for sinensetin (**5**) and syringin (**8**) although both compounds exhibited weak or null DPPH^·^ scavenging [59,60,61]. Conversely, *p*-coumaric acid (**9**), *p*-anisic acid (**10**) and caffeic acid (**11**) are known to possess marked scavenging and chelating antioxidant properties [62,63,64]. These activities could be due to the presence of multiple functional groups, such as hydroxy or methoxy substituents with a carboxyl group as explained by Ivanova et al. [65]. Based on this evidence, the contribution of these compounds to the antioxidant activity of *D. sericea* extract can be hypothesized: this bioactivity could be a result of the subtle interactions of these phytochemicals, although the involvement of the entire phytocomplex cannot be excluded. Further studies could clarify the role of each bioactive constituent in the antioxidant properties of *D. sericea* flowering aerial parts extract.

#### 3.3.2. Cytotoxic Activity

**Figure 6:**
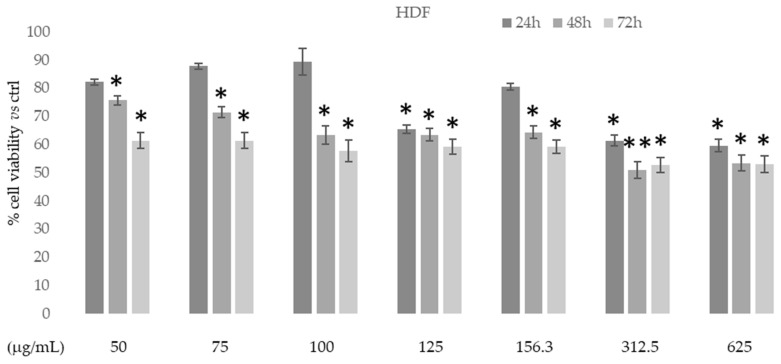
Cytotoxic activity of *Daphne sericea* Vahl flowering aerial parts extract against human fibroblast cell HDF treated for 24, 48, and 72h. The percentage of cell viability (assayed by MTT test) was calculated considering the value of the control as 100%. Results are expressed as the mean value ± SD of quadruplicate determinations from 3 independent experiments. Asterisks indicate a significant reduction in cell viability in treated samples with respect to control cells (one-way ANOVA test, * *p* < 0.05, ** *p* < 0.01).

The in vitro cytotoxic activity of the ethanolic extract of *D. sericea* flowering aerial parts was tested on human fibroblast cell HDF (normal cells) using MTT colorimetric assay at different concentrations and times. Figure 6 shows that in this normal cell line the extract had no cytotoxic effects at all concentrations and times used.

We also evaluated the inhibitory concentration to achieve 50% cell death (IC_50_) for *D. sericea* compound in two human cancer cell lines (MDA-MB-231 and U-87 MG) using MTT assay at the same concentrations of fibroblast cells and after 48h of treatment. The IC_50_ value after 48h of treatment with *Daphne sericea* Vahl found that the ethanolic extract was more effective in the MDA-MB-231 cell line (IC_50_ equal to 75 μg/mL) than in U-87 MG (IC_50_ equal to 150 μg/mL).

**Figure 7:**
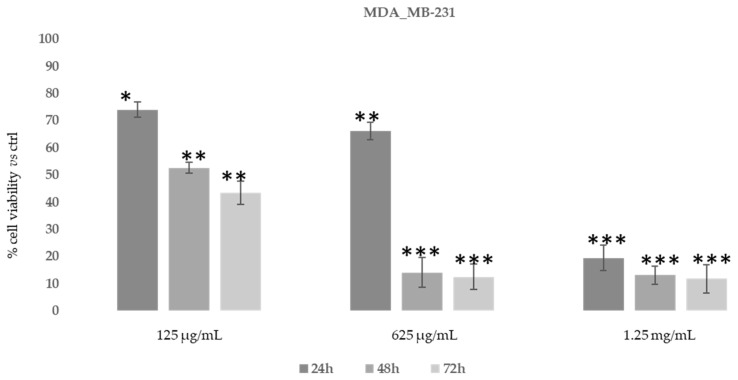
Cytotoxic activity of *Daphne sericea* Vahl flowering aerial parts extract against the MDA-MB-231 cancer cell line treated for 24, 48 and 72 h. The percentage of cell viability (assayed by MTT test) was calculated considering the value of the control as 100%. Results are expressed as the mean value ± SD of quadruplicate determinations from 3 independent experiments. Asterisks indicate a significant reduction in cell viability in treated samples with respect of control cells (one-way ANOVA test, * *p* < 0.05, ** *p* < 0.01; *** *p* < 0.001).

**Figure 8:**
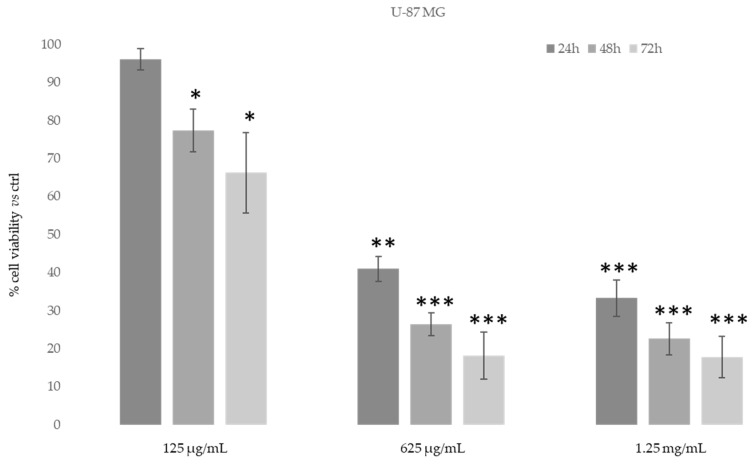
Cytotoxic activity of *Daphne sericea* Vahl flowering aerial parts extract against the U-87 MG cancer cell line treated for 24, 48 and 72 h. The percentage of cell viability (assayed by MTT test) was calculated considering the value of the control as 100%. Results are expressed as the mean value ± SD of quadruplicate determinations from 3 independent experiments. Asterisks indicate a significant reduction in cell viability in treated samples with respect of control cells (one-way ANOVA test, * *p* < 0.05, ** *p* < 0.01; *** *p* < 0.001).

Then, in order to study the anti-proliferative properties of *D. sericea* compound we evaluated the in vitro cytotoxic activity in the same human cancer cell lines using MTT assay (Figure 7 and Figure 8).

As shown in Figure 7, after 48–72 h, the ethanolic extract at the concentration of 625 μg/mL was able to induce an evident reduction in MDA-MB-231 cells’ proliferative capacity (85–90%). However, MDA-MB-231 cells also showed the same reduction after 24 h of treatment with 1.25 mg/mL of *D. sericea* extract. Instead, the U-87 MG cell line revealed to be less drastically susceptible to the anti-proliferative effect of *D. sericea* flowering aerial parts extract both at the concentrations of 625 μg/mL and 1.25 mg/mL after 24, 48 and 72 h of treatment (Figure 8).

Our results also revealed that the ethanolic extract of *D. sericea* flowering aerial parts, in a pathological condition such as cancer, showing important anti-proliferative activities against the two different human cancer cell lines studied. The cytotoxic effects observed in the *D. sericea* Vahl flowering aerial parts extract against the two studied cancer cell lines may also be explained by the total phytocomplex activity. Indeed, most of the identified compounds have already shown relevant antitumor properties against several cell lines [63,66,67,68,69,70,71,72,73]. Among these, sinensetin (**5**) seems likely to be the most responsible for the activity against MDA-MB-231 proliferation since it was observed to decrease the viability of MDA-MB-231 cells in a concentration and time dependent manner [74], whereas the poly-unsaturated fatty acids, *p*-anisic acid (**10**) and caffeic acid (**11**) seem to be the most effective compounds against U87-MG [63,66,75]. Then, these biological activities performed on the same extract showed good effects, in accordance with other previously published results, for what concerns the antioxidant tests and no toxic activities under normal conditions on a human normal fibroblast cell line. These results suggest a potential use of this natural product for integrative therapies thanks to its antioxidant properties. Moreover, the same compound at different concentrations has demonstrated good cytotoxic effects against the two studied cancer cell lines and it may be used in clinical practice as chemosensitizer to enhance the response to conventional therapy in future. Nevertheless, further pharmacological studies on the extract and on the single isolated compounds in this sense are necessary to verify this hypothesis, although the reported results are very promising.

## 4. Conclusions

The first phytochemical analysis of the ethanolic extract of *Daphne sericea* Vahl flowering aerial parts collected in Italy evidenced the presence of eleven compounds, belonging to six different classes of natural compounds. Among these, some were identified in the species and in the family for the first time, whereas the presence of the others confirms the correct botanical classification of the studied specimen as a member of the *Daphne* genus. The biological activities performed on the same extract showed good effects, in accordance with other previously published results, in terms of antioxidant activity and generally good cytotoxic effects against the two studied cancer cell lines. These findings suggest the possibility to list this species among the important medicinally and pharmacologically active *Daphne* species even if further studies are necessary to finally validate this hypothesis.

## Figures and Tables

**Table 1:** The concentration producing a 50% inhibitory response (IC_50_) values of the ethanolic extract obtained from the flowering aerial parts of *Daphne sericea* Vahl and the positive controls in the antioxidant activity assays.

Antioxidant Activity Assays	IC_50_ (Confidence Limits) μg/mL	
	*Daphne sericea* Vahl Flowering Aerial Parts Extract	Positive Control
DPPH^·^ radical scavenger activity	506.8 (404.6–634.9)	16.5 (8.5–133.7) ^a^
ABTS^·+^ radical scavenger activity	11.4 (6.7–19.4)	2.8 (1.7–4.7) ^a^
Ferrous ion chelating activity	143.8 (83.6–247.3)	36.4 (28.1–47.2) ^b^
Ferric ion chelating activity	551.2 (270.6–969.1)	273.0 (198.7–375.1) ^b^

^a^ Trolox. ^b^ Quercetin.

**Table 2:** Pearson correlation coefficient among antioxidant activity assays for the flowering aerial parts extract from *Daphne sericea* Vahl.

	Pearson r (Confidence Limits; R Square)			
	DPPH^·^ scavenger activity	ABTS^·+^ scavenger activity	Ferrous ion chelating activity	Ferric ion chelating activity
DPPH^·^ radical scavenger activity	1	-	-	-
ABTS^·+^ radical scavenger activity	nsc	1	-	-
Ferrous ion chelating activity	0.89 * (0.27–0.99; 0.79)	nsc	1	-
Ferric ion chelating activity	0.99 *** (0.93–0.99; 0.98)	nsc	0.94 ** (0.55–0.99; 0.89)	1

nsc: not significantly correlated. * *p* < 0.05, ** *p* < 0.01 and *** *p* < 0.001, statistically significant correlation (two-tailed *t*-test).

**Table 3:** Amounts of total phenolics and tannins, expressed as tannic acid equivalents (TAE), and flavonoids, expressed as quercetin equivalents (QE), in the ethanolic extract obtained from the flowering aerial parts of *Daphne sericea* Vahl. Data are reported as the average and standard error (SE) of at least six replicates from two experiments.

Compounds	µg/mg Extract
Polyphenols (TAE)	34.1 ± 2.8
Tannins (TAE)	10.1 ± 2.9
Flavonoids (QE)	8.4 ± 0.9

## Data Availability

All the data are available in this work.
